# Feasibility and Reproducibility of Left Ventricular Rotation by Speckle Tracking Echocardiography in Elderly Individuals and the Impact of Different Software

**DOI:** 10.1371/journal.pone.0075098

**Published:** 2013-09-13

**Authors:** Chloe M. Park, Katherine March, Suzanne Williams, Suraj Kukadia, Arjun K. Ghosh, Siana Jones, Therese Tillin, Nish Chaturvedi, Alun D. Hughes

**Affiliations:** International Centre for Circulatory Health, Imperial College London, London, United Kingdom; National Institutes of Health, United States of America

## Abstract

**Background:**

Changes in ventricular rotation measured by two-dimensional speckle tracking echocardiography (2DSTE) are early indicators of cardiac disease. Data on the clinical feasibility of this important measure are scarce and there is no information on the comparability of different software versions. We assessed the feasibility, reproducibility and within patient temporal variability of 2DSTE in a large community based sample of older adults. We additionally compared 2DSTE results to those generated by 3DSTE.

**Methods and results:**

1408 participants underwent transthoracic echocardiography. Using Philips Qlab 8.1 peak LV rotation at either the base or the apex was analysable in 432 (31%) participants. Peak twist measurements were achieved in 274 (20%) participants. 66 participants were randomly selected for the reproducibility study. 20 additional participants had scans 4–6 weeks apart for temporal variability and 3D echocardiography to assess the agreement between 2DSTE and 3DSTE. Reproducibility was evaluated using the intraclass coefficient of correlation (ICC). Better reproducibility for rotation and twist were obtained when measured at the endocardium, and when using more recent software versions, Peak twist and rotation were significantly different using two versions of the same software. Agreement with 3DSTE was better using newer software.

**Conclusion:**

Feasibility of 2DSTE is low in this cohort of elderly individuals severely limiting its utility in clinical settings. However if high quality images can be acquired assessment of ventricular rotation by 2DSTE is reproducible. Caution should be taken when comparing measurements of ventricular rotation by software from different vendors or different versions of software from the same vendor.

## Introduction

With population ageing, dysfunction of the left ventricle (LV) is an increasingly common manifestation of cardiovascular disease, and a key contributor to morbidity and mortality [1]. Mechanisms underlying pathways to LV dysfunction are poorly understood and our understanding of the early stages of dysfunction may be enhanced by the assessment of LV rotation and twist using echocardiographic methods. However while research on other measures of LV deformation such as strain analysis are thriving, measurement of LV rotation is often neglected.

Myocardial fibres are arranged in layers of oppositely wound helices [2]. The subendocardial right-handed helix transforms into a left-handed helix in the subepicardium. When viewed from the apex, contraction of epicardial fibres produces anti-clockwise rotation at the apex and clockwise rotation at the base [3]–[5]. Contraction of the endocardial fibres results in rotations in the opposite direction, however the higher torque developed in the subepicardial fibres dominates the overall direction of rotation [5], [6]. This alignment of fibre architecture of the LV serves to equalise transmural stress and strain, generating an energy efficient system [6]. In diastole, potential energy from systolic twist is discharged, contributing to LV suction and enhancing diastolic filling. Pathophysiological processes that alter LV fibre architecture will result in uneven stresses and a higher myocardial oxygen demand. Altered rotational mechanics of the LV have previously been described in aging [7], diastolic dysfunction [8], congestive heart failure [9], dilated cardiomyopathy [10], and many more disease states[11]–[13]. The precise changes in LV rotational patterns are specific to individual pathologies [13] but, abnormalities of rotation may be useful as early indicators of cardiac disease[14]–[17].

Two dimensional speckle tracking echocardiography (2DSTE) is becoming a popular modality for quantifying LV rotation and twist. It has been validated against cardiac magnetic resonance (MR) [18], [19] and the reproducibility of 2DSTE is reported to be high [20], [21]. Previous reproducibility studies have largely been performed in selected younger individuals; largely in the absence of subclinical disease, which itself might influence measurement reproducibility in older individuals. In addition, newer post-processing packages may offer substantial gains in accuracy and reproducibility. We therefore aimed to primarily assess the feasibility of this technique in a large community-based cohort of older individuals. Secondly we tested the inter- and intra-observer reproducibility and repeatability over time, of rotation measured by 2DSTE, using an established and a recently developed version of QLab software. Furthermore, we investigated the precision of 2DSTE by comparing peak rotational and twist results to those from newly released 3DSTE software.

## Methods

### Ethics Statement

The study was approved by the Imperial College London and Imperial College healthcare NHS trust committee on human research. All clinical investigations were conducted according to the principles expressed in the Declaration of Helsinki. All participants gave written informed consent to participate.

### Study Population

A total of 1436 individuals attended the Southall and Brent Revisited (SABRE) clinic between June 2008 and March 2011. SABRE is a population-based tri-ethnic longitudinal cohort of several thousand individuals (average age 69.6±6.2 years) [22]. Participants were excluded only if they had severe co-morbidity sufficient to preclude a one day clinic visit, or were unable to provide written informed consent.

### Clinic Measurements

Participants attended the study clinic at St Mary’s Hospital, London following an overnight fast. During their visit, resting blood pressure, ECG and anthropometric measurements were recorded and fasting blood samples were taken. Resting blood pressure was measured in the sitting position, 3 left brachial blood pressure measurements were recorded, at 2 minute intervals. The second and third recordings were averaged. Height was measured using a stadiometer and weight was measured using electronic bioimpedance scales. Cardiovascular and diabetes related events were identified from primary-care medical review and participant questionnaire. Diabetes was recorded if previously diagnosed by a doctor or newly diagnosed from the clinic oral glucose tolerance test. Probable CHD was defined as presence of or a history of doctor diagnosed angina or heart attack or presence of the following ECG findings (Minnesota coded) [23]: major Q wave(code 1_1–2_ or borderline Q wave (code 1_3_) ST depression or elevation (4_1–3_), deep or moderate T wave inversion (code 5 _1–3_)_or left bundle branch block(code 7_1_). Coronary interventions were defined as coronary angioplasty or stents or coronary artery bypass grafting identified from primary care medical record review or participant report.

### 2D Echocardiography

Echocardiography was performed on 1408 SABRE clinic participants (average age 69.6±6.2 years) by two experienced sonographers on a Philips iE33 ultrasound machine equipped with a 5.0–1.0 phased array transducer (S5-1). Two-dimensional grey-scale images were acquired in the left lateral decubitus position. Parasternal short-axis views were obtained at the LV base at the level of the mitral valve and at the LV apex. The sonographers underwent specific training to acquire optimal images with the objective of performing rotational analysis. Great care was taken to ensure that all images were as circular as possible and at the exact level of the ‘true’ apex (immediately proximal to luminal obliteration) and base (ensuring that the tips of the mitral valve are visible). This often required re-positioning of the transducer in-between base and apex image acquisition. Single cardiac cycle images were acquired at each LV level at a frame-rate of 60–80 frames per second (FPS) and at an increased gain.

### 2DSTE Feasibility

All short axis images were transferred to a PC containing Philips 2DSTE software Qlab 8.1. Adequacy of tracking was assessed using an innovative tracking quality tool based on a user defined threshold in Qlab 8.1.

### 2DSTE Reproducibility

All echocardiograms were stored on DVDs (∼6/7 cases per DVD) and for the rotation reproducibility element of the study 10 DVDs were randomly selected. These DVDs contained a total of 66 echocardiograms to be analysed, this sample size adhered to the statistical note on how to decide the sample size for a repeatability study [24]. Images from these 10 DVDS were transferred to 2 PCs for analysis, one using Philips Qlab 2DSTE software 7.0 and the second Qlab 8.1. A total of 40 of the 66 echocardiograms randomly selected had adequate images at either or both the apical and basal level of the LV to be analysed using this technique (60.6% success rate). Adequacy was decided as described above. LV rotation reproducibility was assessed in these 40 individuals. LV short axis images at the level of the base and/or apex were acquired and a total of 25 images at the apex and 25 images at the base were of a high enough standard to analyse using 2DSTE. The reproducibility of transmural torsion was also calculated and reported on these individuals. LV twist reproducibility was assessed in 15 of these participants. These individuals had adequate images at both apex and base, which enabled the calculation of twist.

Long-term reproducibility of both LV rotation and twist was performed on an additional 20 participants who were known to have adequate images at both the base and apex after their first visit (3 of these individuals were from the 10 randomly selected DVDs mentioned above).These 20 individuals had repeated measurements at both the apex and base made 4–6 weeks after their initial visit. Baseline characteristics of all participants for both the short term and long term reproducibility are presented in table 1 (total n = 57).

**Table 1:** Characteristics of SABRE participants split by gender and feasibility of LV peak rotation measures.

	Men				Women			
	No rotational measures	Rotation at baseor apex	Rotation at bothbase and apex	Anova p	No rotationalmeasures	Rotation at baseor apex	Rotation at bothbase and apex	Anova p
n	534(50)	326(31)	206(19)		168(49)	106(31)	68(20)	
Age, y	70.1±6.3	69.7±6.0	69.2±6.2	0.08	69.7±6.4	69.2±6.0	67.7±5.9*	0.03
Height, cm	171±8	171±7	171±3	0.4	158±7	158±6	159±7	0.3
Weight, kg	81.0±6	79.1±13	76.6±12**	0.0001	74.8±15	70.7±16*	69.4±14**	0.007
Body Mass Index, kg/m^2^	27.7±5	27.0±4*	26.1±3**	<0.0001	29.9±6	28.4±6*	27.1±5**	0.0005
Waist to hip ratio	1.00±0.07	1.00±0.06	0.98±0.06**	0.0003	0.93±0.08	0.92±0.08	0.91±0.08	0.2
Heart Rate, beats/min	68±12	67±13	66±12*	0.02	70±12	70±11	69±11	0.9
Systolic BP, mmHg	140±16	143±20	141±17	0.3	139±20	137±18	137±16	0.6
Diastolic BP, mmHg	82±10	82±10	82±10	0.9	79±9	78±9	82±10	0.1
BP treatment, n (%)	365(68)	215 (66)	130 (63)	0.4	112(67)	69(65)	33(49)*	0.03
Diabetes, n (%)	173(32)	100(31)	59(29)	0.6	56(33)	31(29)	18(27)	0.5
Left Ventricle Mass (g)	190±54	190±50	179±46*	0.03	160±42	149±42*	150±40	0.04
Relative Wall Thickening	0.46±0.09	0.46±0.08	0.46±0.07	0.9	0.47±0.09	0.48±0.09	0.50±0.09	0.1
Probable CAD, n (%)	154 (29)	77(24)	53(26)	0.4	40(24)	14(13)*	6(9)**	0.009
Coronary intervention, n (%)	103(19)	53(16)	29(14)	0.2	16(10)	11(10)	1(2)*	0.08

Data are mean±SD for numerical data and n (%) for categorical data.

* = p<0.05.

** = p<0.01 compared with individuals that had no rotation measurements by post hoc test following ANOVA. BP, blood pressure; CAD coronary artery disease.

### 3D Echocardiography

3D echocardiography was performed on a subset of 20 participants. 3D full volume sets were acquired immediately after 2D echocardiography by the same sonographer on a Philips iE33 ultrasound machine. 3D full volume sets were acquired from the apical 4 chamber view with a matrix array transducer (X3). Data sets were acquired over 4 cardiac cycles during held respiration in the wide-angled acquisition mode (93°×80°). Four sub volumes are obtained to form a full-volume set. The images were transferred to a PC for analysis using Tomtec 4D LV-Analysis (TomTec Imaging Systems, Munich, Germany).

### Data Analysis

#### 2DSTE

LV short-axis images were analysed using Philips Qlab Advanced Tissue Motion Quantification (TMQA) software version 7.0 and Cardiac Motion Analysis (CMA) software version 8.1.

**Figure 1:**
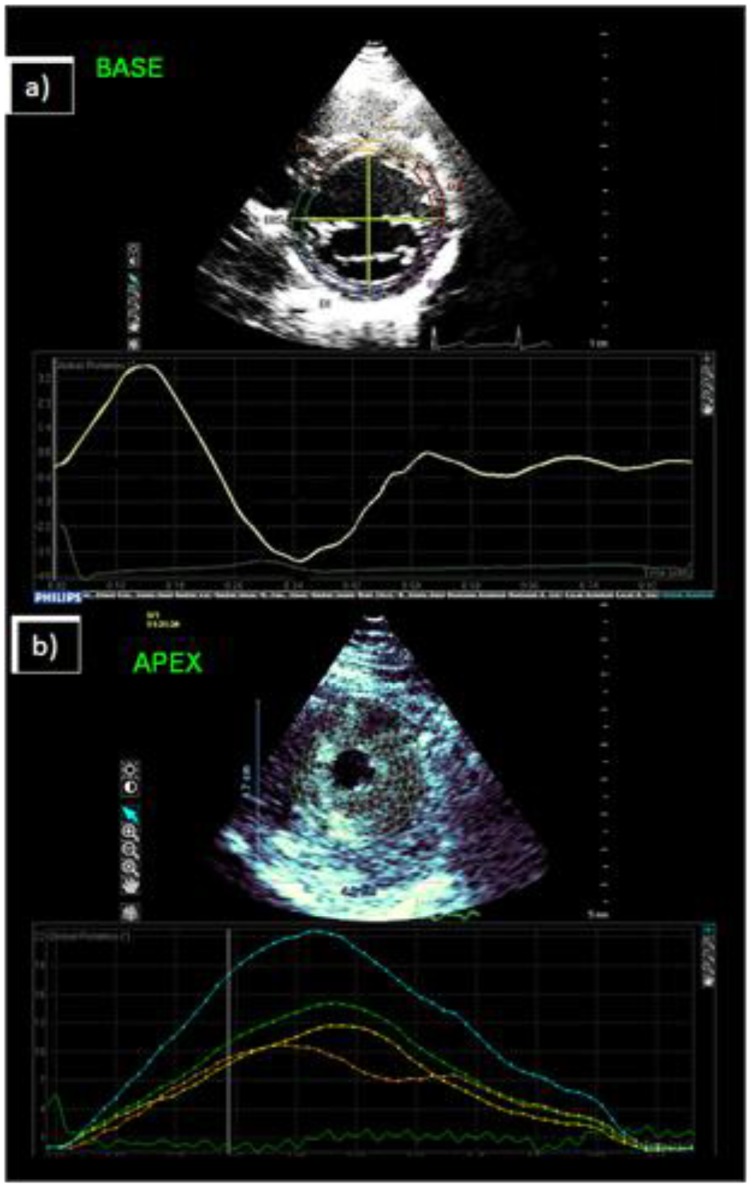
Speckle tracking echocardiography examples. **A. Qlab 7.0**, this is an example of speckle tracking at the LV base level. A single layer of tracking points is placed on either the endocardium or epicardium ∼60° away from each other. **B. Qlab 8.1,** an example of the LV short axis at the apex level. A mesh tracks all layers of the myocardium simultaneously.

One feature of TMQA is the option to track the endocardium and epicardium simultaneously. However following preliminary studies and the observations of Van Dalen *et al*. [20] who demonstrated that concentrating on one layer of tracking points at a time is a more robust method than tracking two layers simultaneously, we limited our analysis using Qlab 7.0 to one myocardial layer at a time. Six tracking points were placed manually in the endocardium on the end-diastolic frame of each short-axis image ∼60° away from each other. Qlab tracks the chosen points on a frame-by-frame basis and generates rotational profiles. If any points showed poor tracking by visual assessment they were changed manually on the end-diastolic frame. Once, and only if, all points showed good tracking were the data exported to a Microsoft Excel spreadsheet for further analysis. If good tracking of all points was not achieved the image was graded as unsatisfactory and not included in the analysis. The same method was used to calculate epicardial rotation **Figure 1A**. Intra-observer reproducibility was assessed by the same reader repeating all analyses one month later. Inter-observer reproducibility was assessed by comparison with a second reader who had no knowledge of the first set of results. Long-term test-retest variability was assessed by a single reader masked to participant identity.

Philips has more recently developed an updated version of the Qlab software (Qlab 8.1). The speckle tracking algorithm has been altered and the TMQA software has been replaced with the CMA software. Instead of tracking 6 points placed ∼60° away from each other CMQ uses a mesh of tracking points across the entire wall as demonstrated in **Figure 1B**. The CMQ tracking mesh was positioned on the frame when delineation of the myocardium was optimal. All LV short-axis analyses for intra-observer reproducibility and long-term variation were repeated using Qlab 8.1 CMQ to determine if the new software was a) compatible with Qlab 7.0 and b) more capable of successfully tracking the endocardium and epicardium simultaneously.

#### 3DSTE

Tomtec 4D LV-Analysis is vendor independent software that can analyse real time 3D full volume sets to generate global parameters of rotation and twist. The software achieves this by applying an advanced automatic contour finding algorithm combined with 3D speckle tracking to the endocardial border in 3 planes (apical 2-, 4-, and “3-” chamber views). The results acquired using 3D STE were compared to those from Qlab 7.0 and 8.1.

### Statistical Analysis

Statistical analysis was performed using STATA (version 12). Data are presented as mean (95% CI). Method comparison between QLab 7.0 and 8.1 and Tomtec 4D-LV analysis were made as described by Bland and Altman [25]. Statistical comparisons between epicardial and endocardial layers and between QLab 7.0 and 8.1 were made using linear mixed model analyses. Reproducibility was assessed using the following measures: mean difference±SD_diff,_ intra-class coefficient of correlation (ICC), [26]. An ICC ≥0.75 was deemed excellent, >0.40 to <0.75 fair and ≤0.40 poor agreement [27].

## Results

### Feasibility

50% of male participants and 49% of female participants did not have adequate LV short axis images to analyse at either the base or the apex. 31% of both men and women had adequate images at one site, either the base or the apex and 19% of men and 20% of women had adequate images at both the apex and the base, allowing the calculation of LV twist.

**Table 1** presents the gender specific characteristics of these sets of participants.

Blood pressure, height, diabetes status and LV shape (relative wall thickness) did not differ between groups, however individuals in whom the calculation of rotation was not possible were significantly heavier compared to those with measurements at either the base or the apex. Men with rotational measures at both the base and apex were significantly lighter with lower heart rates and smaller LV mass. Women with rotational measures at both the base and apex were significantly younger and lighter on less blood pressure treatment, with smaller LV mass and less probable coronary artery disease and coronary interventions.

### Reproducibilit*y*

#### Characteristics

The participants involved in the reproducibility study were on average 70.1±6.2****yrs with 73% being male. Resting blood pressures were within the normal range; however 63% were on hypertensive medications. There was little clinical evidence of coronary disease (Table 2).

**Table 2:** Baseline characteristics of participants in the reproducibility studies.

Characteristic	
n	57
Age, y	70.1±6.2
Male, n (%)	42 (73)
Height, cm	169±9
Weight, kg	74.7±12
Body Mass Index, kg/m^2^	26.3±3.6
Heart Rate, beats/min	67±12
Systolic Blood Pressure, mmHg	136±16
Diastolic Blood Pressure, mmHg	76±10
Myocardial Infarction, n (%)	3 (5)
Coronary Artery Bypass Graft, n (%)	4 (7)
Heart Failure, n (%)	1 (1.7)
Angioplasty, n (%)	5 (9)
Hypertension, n (%)	36 (63)
Diabetes, n (%)	15 (26)

Data are mean±SD or n (%).

#### Comparison of Qlab 7.0 and 8.1

All comparisons of Qlab 7.0 and 8.1 are presented in Table 3. Measures of peak LV twist and rotation were significantly higher when measured using Qlab 7.0 compared to Qlab 8.1. Endocardial measures were consistently greater than epicardial measures using either software version. Agreement between Qlab 7.0 and 8.1 was excellent for peak apical endocardial rotation (ICC = 0.79) but fair for all other measurements (apical epicardium ICC = 0.45, base endocardium ICC = 0.50 and base epicardium ICC = 0.44).

**Table 3:** Comparison of Qlab 7.0 and 8.1.

		Qlab 7.0			Qlab 8.1			Difference betweenQlab 7.0 and 8.1	
		Endocardium	Epicardium	P	Endocardium	Epicardium	P	Endocardium P	Epicardium P
**Peak Twist (°)**	**(n = 26)**	14.38 (12.6,16.1)	9.05 (7.86,10.3)	<0.0001	11.22 (10.1,12.4)	6.31 (5.4,7.2)	<0.0001	0.0005	0.0001
**Peak rotation (°)**	**Apex (n = 42)**	9.04 (7.8,10.3)	5.4 (4.5,6.3)	<0.0001	7.29 (6.1,8.5)	4.15 (3.5,5.0)	<0.0001	0.0001	0.0002
	**Base (n = 41)**	−6.18 (−6.9,−5.5)	−4.06 (−4.7,−3.4)	<0.0001	−4.69 (−5.3,−4.0)	−2.88 (−3.4,−2.4)	<0.0001	<0.0001	<0.0001
**t_max_ (s)**	**Apex (n = 42)**	0.38 (0.36,0.40)	0.38 (0.36,0.40)	0.74	0.38 (0.36,0.40)	0.37 (0.36,0.40)	0.8	0.72	0.78
	**Base (n = 41)**	0.37 (0.35,0.39)	0.38 (0.38,0.39)	0.77	0.37 (0.35,0.39)	0.37 (0.35,0.40)	0.79	0.91	0.46

Data are average values (95% confidence intervals) for endocardial and epicardial peak twist, rotation and time to peak rotation (t_max_) measured at the left ventricle apex and base.

#### Intra-observer reproducibility of both Qlab 7.0 and 8.1

**Figure 2:**
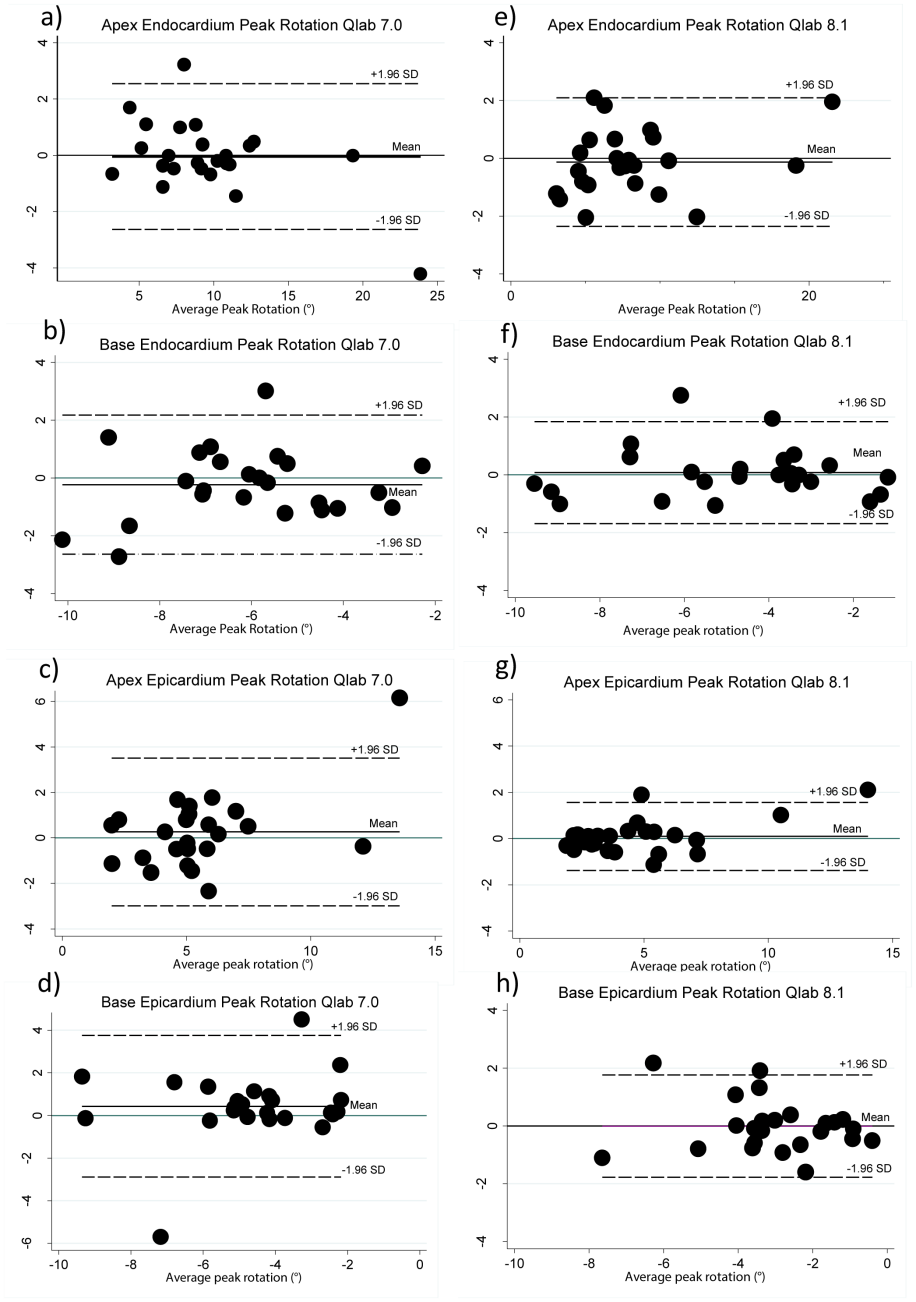
Qlab 7.0 and 8.1 Intra-observer agreement. Bland & Altman plots a) Qlab 7.0 apex peak rotation at the endocardium b) Qlab 7.0 base peak rotation at the endocardium c) Qlab 7.0 apex peak rotation at the epicardium d) Qlab 7.0 base peak rotation at the epicardium. e) Qlab 8.0 apex peak rotation at the endocardium f) Qlab 8.1 base peak rotation at the endocardium g) Qlab 8.1 apex peak rotation at the epicardium h) Qlab 8.1 base peak rotation at the epicardium.

Intra-observer reproducibility was high for peak systolic twist using 8.1, yet poor using Qlab 7.0 (Figure 2, Table 4). Long term reproducibility for peak twist was greater at the endocardium (ICC = 0.80) than the epicardium (ICC = 0.54) using Qlab 8.1.

**Table 4:** Qlab 7.0 and 8.1 Intra-observer reproducibility.

		Qlab 7.0		Qlab 8.1	
		Endocardium	Epicardium	Endocardium	Epicardium
**Repeat results**					
**Peak Twist (°)**	**(n = 15)**	1.3±3.4 (0.46)	1.5±2.6 (0.33)	0.9±0.07 (0.87)	−0.2±1.1 (0.90)
**Peak Rotation (°)**	**Apex (n = 25)**	0.0±1.3 (0.96)	0.3±1.7 (0.82)	−0.1±1.1 (0.97)	0.1±0.8 (0.96)
	**Base (n = 25)**	−0.2±1.2 (0.82)	0.0±4.8 (0.68)	0.1±0.9 (0.93)	0.0±0.9 (0.87)
**t_max_ (s)**	**Apex (n = 25)**	0.00±0.02 (0.97)	0.00±0.02 (0.96)	−0.0±0.04 (0.78)	0.00±0.02 (0.92)
	**Base (n = 25)**	0.01±0.03 (0.86)	−0.01±0.05 (0.66)	−0.01±0.05 (0.70)	0.01±0.06 (0.70)
**Long-term test-retest results**					
**Peak Twist (°)**	**(n = 20)**	3.67±3.96 (0.44)	2.96±2.59 (0.38)	0.48±1.66 (0.80)	−0.10±2.1 (0.54)
**Peak Rotation (°)**	**Apex (n = 20)**	−1.3±2.6 (0.32)	−1.6±2.1 (0.45)	0.36±1.75 (0.63)	0.03±1.9 (0.29)
	**Base (n = 20)**	2.4±3.2 (0.42)	1.3±2.0 (0.23)	0.11±1.45 (0.61)	0.13±1.2 (0.55)
**t_max_ (s)**	**Apex (n = 20)**	−0.01±0.06 (0.61)	−0.01±0.06 (0.53)	−0.02±0.07 (0.25)	−0.02±0.08 (0.22)
	**Base (n = 20)**	0.01±0.06 (0.55)	0.02±0.07 (0.35)	−0.00±0.07 (0.51)	−0.01±0.06 (0.42)
**Peak transmural Torsion(°)**	**Apex (n = 25)**	NA		−.022±0.9 (0.89)	
	**Base (n = 25)**			−0.35±1.4 (0.50)	
**t_max_ (s)**	**Apex (n = 25)**			0.01±0.03 (0.89)	
	**Base (n = 25)**			0.01±0.05(0.81)	

Reproducibility of endocardial and epicardial peak twist, rotation and time to peak rotation measured at the left ventricle apex and the base. Data are average difference ±SDdiff (ICC, concordance correlation coefficient).

Apical peak rotation showed excellent intra- -observer reproducibility at both the endocardium and epicardium (Table 4 ). Long-term reproducibility was less good, and was best for endocardial peak rotation measures using Qlab 8.1 as shown in Table 4. Basal peak rotation was more reproducible at the endocardium; with Qlab 8.1 performing better than Qlab 7.0, specifically for long-term reproducibility. Figure 2 shows all Bland & Altman plots for Qlab 7.0 and 8.1 Intra-observer agreements.

Intra- observer agreement for t_max_ was excellent in both layers when measured at the apex and the base. No significant time difference was observed for time to peak rotation (t_max_) measured at the endocardium compared to the epicardium at either the apex or base (Table 3).

Cardiac cycle duration variability was satisfactory in between the two long-term visits (33±98 ms, ICC = 0.67). Long-term variability for t_max_ was poorer at both layers of the base and apex (Table 4) especially for time to peak apical rotation when using Qlab 8.1.

#### Qlab 8.1 Transmural torsion reproducibility

Transmural torsion could not be calculated using Qlab 7.0 as endocardial and epicardial rotation could not be reliably assessed simultaneously. Using Qlab 8.1 intra-observer agreement for peak transmural torsion was excellent at the apex and satisfactory at the base. Intra-observer t_max_ was also excellent at both the apex and base. (Table 4).

#### Intra-observer reproducibility of Qlab 7.0

Apical rotation and t_max_ showed excellent inter-observer reproducibility at both the endocardium and epicardium. Inter-observer reproducibility for basal rotation was higher at the endocardium than the epicardium (Table 5).

**Table 5:** Qlab 7.0 Inter-observer reproducibility.

		Endocardium	Epicardium
**Peak Twist (°)**	**(n = 8)**	−1.9±2.9 (0.36)	2.5±5.1 (0.13)
**Peak rotation (°)**	**Apex (n = 25)**	−0.6±2.1 (0.89)	−0.3±1.4 (0.79)
	**Base (n = 25)**	−0.2±2.5 (0.75)	−0.01±3.2 (0.34)
**t_max_ (s)**	**Apex (n = 25)**	−0.00±0.07 (0.66)	0.01±0.06 (0.59)
	**Base (n = 25)**	0.01±0.04 (0.84)	0.01±0.05 (0.66)

Reproducibility data are average difference±SDdiff (ICC); t_max_, time to left ventricular peak systolic rotation; ICC, concordance correlation coefficient.

#### Comparison of 2DSTE (Qlab) and 3DSTE (Tomtec)

**Figure 3:**
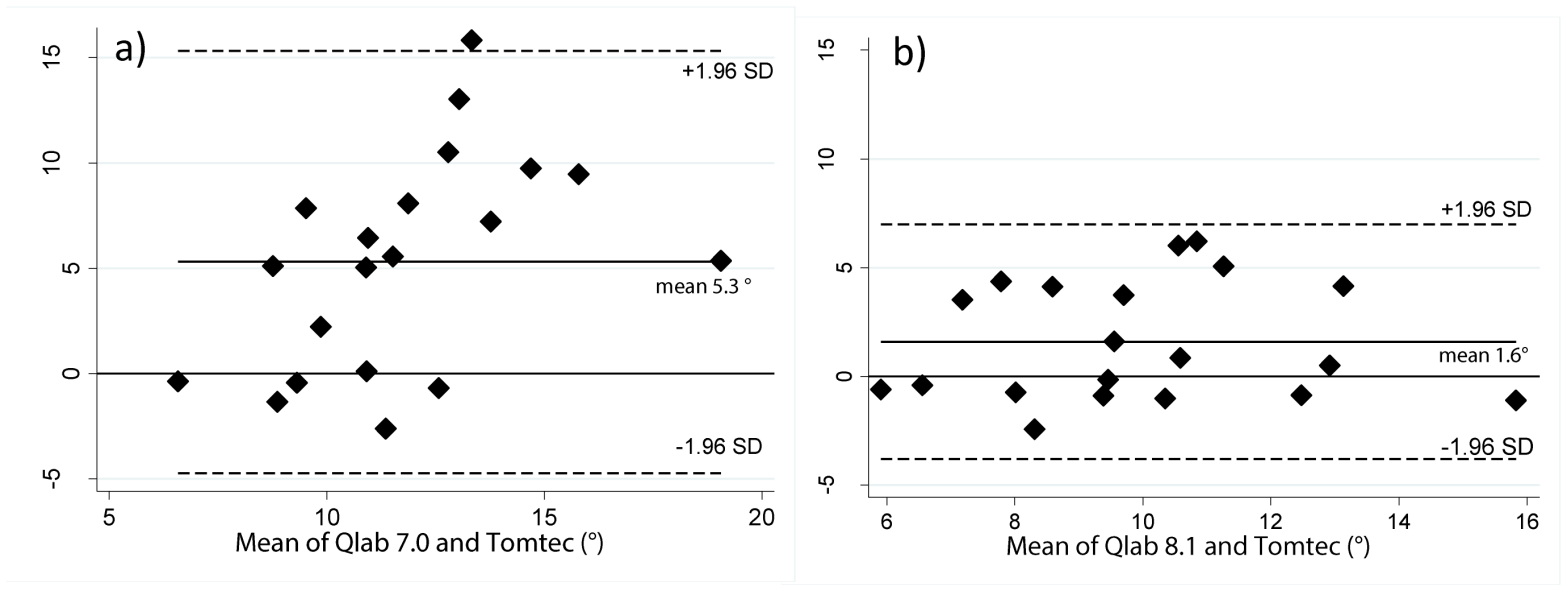
Vendor comparison Bland-Altman plots. Comparison of peak twist a) Qlab 7.0 and Tomtec LV analysis b) Qlab 8.1 and Tomtec LV analysis.

Comparison of 2DSTE using both Qlab 7.0 and 8.1 with 3DSTE using TomTec 4D-LV analysis was also performed. Table 6 presents the calculated average differences and 95% limits of agreement for peak apical and basal rotation and twist at the endocardium. All 3DSTE measurements were in better agreement with Qlab 8.1 measurements than with Qlab 7.0. The comparisons of peak LV twist are shown in Figure 3.

**Table 6:** Comparison of 2DSTE and 3DSTE.

	Qlab 7.0 V Tomtec 4D (n = 20)		Qlab 8.1 V Tomtec 4D (n = 20)	
	Difference±SD	95% LOA	Difference±SD	95% LOA
**Peak apical Rotation (°)**	2.3±3.8	−5.1,9.6	0.2±2.4	−4.5,5.0
**Peak basal Rotation (°)**	−3.03±2.8	−8.5,2.4	−1.4±2.1	−5.4,2.7
**Peak twist (°)**	5.3±5.1	−4.7,15.3	1.6±2.8	−3.8,7.0

Data are mean difference±SD and 95% limit of agreement (LOA).

## Discussion

The results presented in this study demonstrate that feasibility of 2DSTE is low in this cohort of elderly individuals probably making this an unacceptable tool for regular cardiac assessment in clinical settings. However when adequate images are acquired 2DSTE is generally highly reproducible for assessing left ventricular rotation, even in an older population. Age, weight, heart rate, LV mass and presence of probable coronary artery disease have significant effects on the success rate of this technique.

Furthermore we show that different versions of the same analysis software (QLab) resulted in significantly different estimates of apical and basal rotation. This is an important consideration when comparing results, even if they have been analysed using software from the same manufacturer. Serial STE studies must be performed using the same instrument and same version of the analytical software. Better standardisation of software between manufacturers and between software releases is desirable.

When using Philips Qlab version 7.0 the most reproducible method for quantifying rotation is by tracking the endocardium only. This is true for both apical and basal short-axis slices. Epicardial tracking was significantly more reproducible at the apex compared to the base. This can be explained by the apex being relatively fixed during the cardiac cycle whereas the base moves toward the apex generating out-of plane motion. The improved reproducibly at the endocardium compared to the epicardium has been attributed to signal saturation and problems related to tracking of non-moving speckles outside the heart [20].

The new algorithm and mesh developed for Qlab 8.1 tracks the entire myocardium and allowed successful, reproducible tracking of the endocardium and epicardium simultaneously. The reproducibility of all endocardial measures of rotation were similar to those using Qlab 7.0, however epicardial measures were considerably more reproducible using this method and there was little difference in reproducibility between layers. By accurately and reproducibly tracking two layers simultaneously Qlab 8.1 CMQ can potentially be utilised to differentiate between diseases which affect the myocardial layers in distinct ways [28]–[30] and permits more reliable assessment of torsion [31].

Agreement between measures derived from repeat echocardiography at an interval of several weeks was high when analysed with Qlab 8.1. Using both Qlab 7.0 and Qlab 8.1 long-term variability tended to be better at the apex compared to the base and at the endocardium compared to the epicardium. The long-term test-retest results agree with the observations of intra-observer reproducibility; Qlab 8.1 has less variability than Qlab 7.0.

### Comparison with Previous 2DSTE Work

The feasibility of 2DSTE has previously been reported at 66.6%; however the average age of individuals in this earlier study was at least 20 years younger than that of the SABRE participants [20]. Peak rotation and twist values in the literature differ substantially depending on the characteristics of the group studied and the technique used [7], [32], [33] but the Qlab 8.1 values presented in the current study are similar to those reported by Goffinet et al. [21] who used Qlab 6.0 to look at endocardial and epicardial rotation in older individuals with a range of cardiac complications.

Qlab 7.0 consistently gave higher values for peak rotation than Qlab 8.1. There are no other studies currently published using Qlab 8.1, however data reported by several other groups using other systems such as Echopac [34] agree well with Qlab 8.1, especially for peak rotation at the base (−4.7±2.6° compared to −4.7 (−5.3,−4.0)° in the current study), the results of which are also similar to MRI-estimates [19], [21].

### 2DSTE and 3DSTE Comparison

Due to a lack of compatibility between file formats and/or a lack of rotational measures generated by certain software the comparison of 2DSTE rotational results generated from different manufacturers was not possible. However Tomtec’s 4D LV analysis software is vendor independent and can be used to calculate endocardial LV rotation and twist using 3DSTE. The values generated by 3DSTE were consistently closer to those generated by Qlab 8.1 than those from Qlab 7.0.

### Limitations

The greatest limitation to STE is the difficulty of obtaining satisfactory images in all individuals. Less than 20% of individuals had images of a high enough quality to calculate all parameters including LV twist. A limitation specific to this study is the focus on the feasibility and reproducibility of LV rotation during systole. Future work should involve investigating LV untwisting during diastole and other aspects of LV mechanics that can be assessed using STE including strain analysis. 2DSTE and 3DSTE comparisons should also be approached with caution as the 3DSTE independent software analysed DICOM images rather than the proprietary raw data that the 2DSTE Philips software uses. It also remains unclear whether current 3DSTE techniques for twist analysis are superior to 2DSTE.

### Clinical Implications

As mentioned in the introduction, LV rotation is a neglected aspect of LV deformation analyses. The only previous feasibility and reproducibility studies have been performed in selected younger individuals; largely in the absence of subclinical disease. The importance of this study is that it shows that 2DSTE is not yet ready be used to regularly assess LV rotation in older individuals. This is because older individuals are often overweight and do not have optimal echogenic windows. Hopefully in the future the quality of both imaging and analysis software will increase as this age group could greatly benefit from the detection of small changes in LV performance that go undetected by conventional echocardiography measures.

### Conclusions

Assessment of ventricular rotation by 2DSTE is reproducible. However adequate images can only be achieved in 50% of older individuals. In addition, different vendor software and different versions from the same manufacturer give significantly different estimates of rotation and twist. 2DSTE may prove to be a valuable tool in the echocardiographic assessment of ventricular function in the future but at the moment the low feasibility of this technique would appear to limit its widespread use in clinical settings.
